# Supplementary material for: A large-scale whole-genome sequencing analysis reveals highly specific genome editing by both Cas9 and Cpf1 (Cas12a) nucleases in rice
Source: Genome Biol. 2018 Jul 4;19:84. doi: 10.1186/s13059-018-1458-5 (PMC6031188; doi:10.1186/s13059-018-1458-5)
Supplement: Supplementary file 1 — Figures S1–S9. Supplemental Figures- part I. (PPTX 12111 kb) [file 13059_2018_1458_MOESM1_ESM.pptx]

## Slide 1
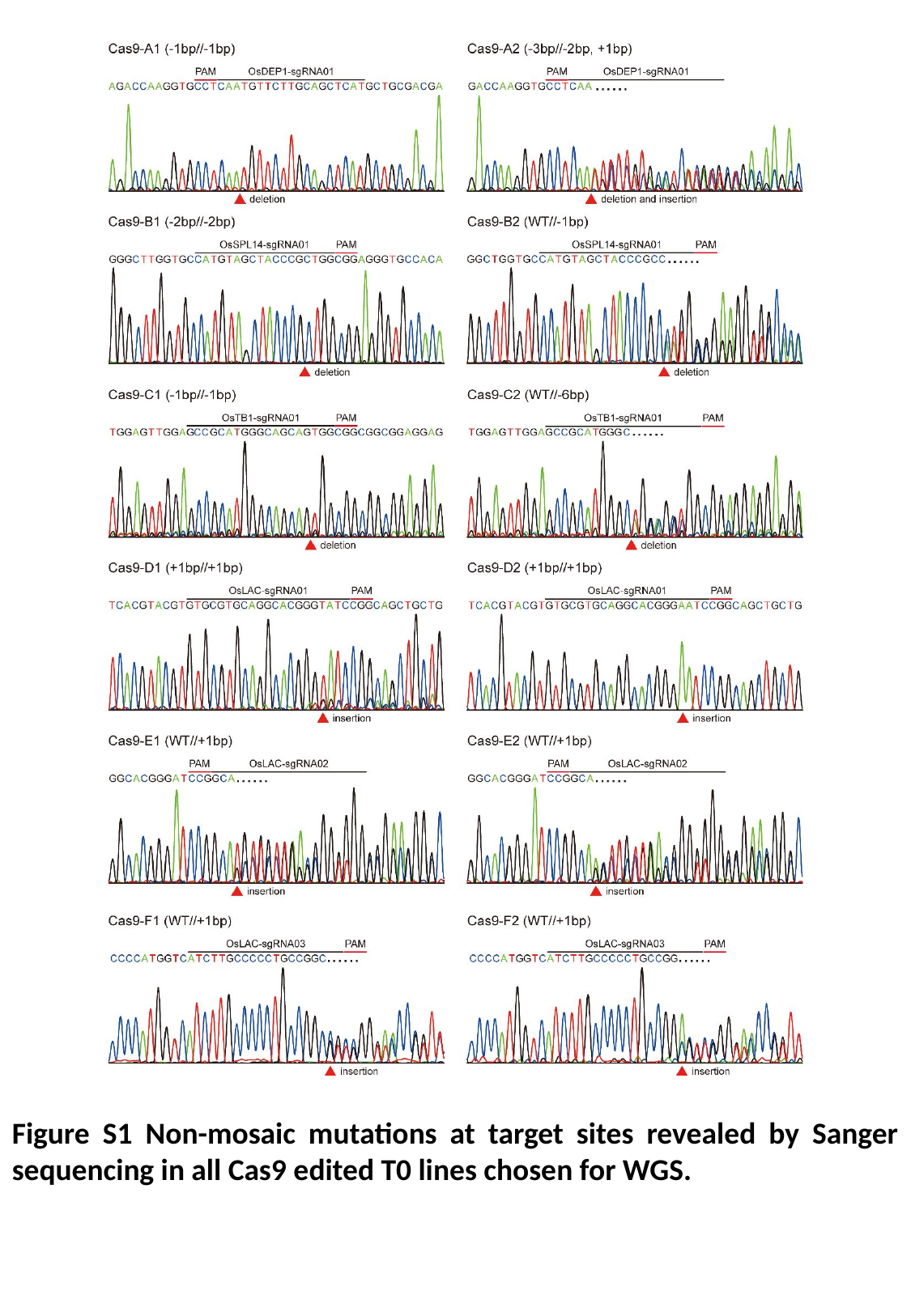

Figure S1 Non-mosaic mutations at target sites revealed by Sanger sequencing in all Cas9 edited T0 lines chosen for WGS.

## Slide 2
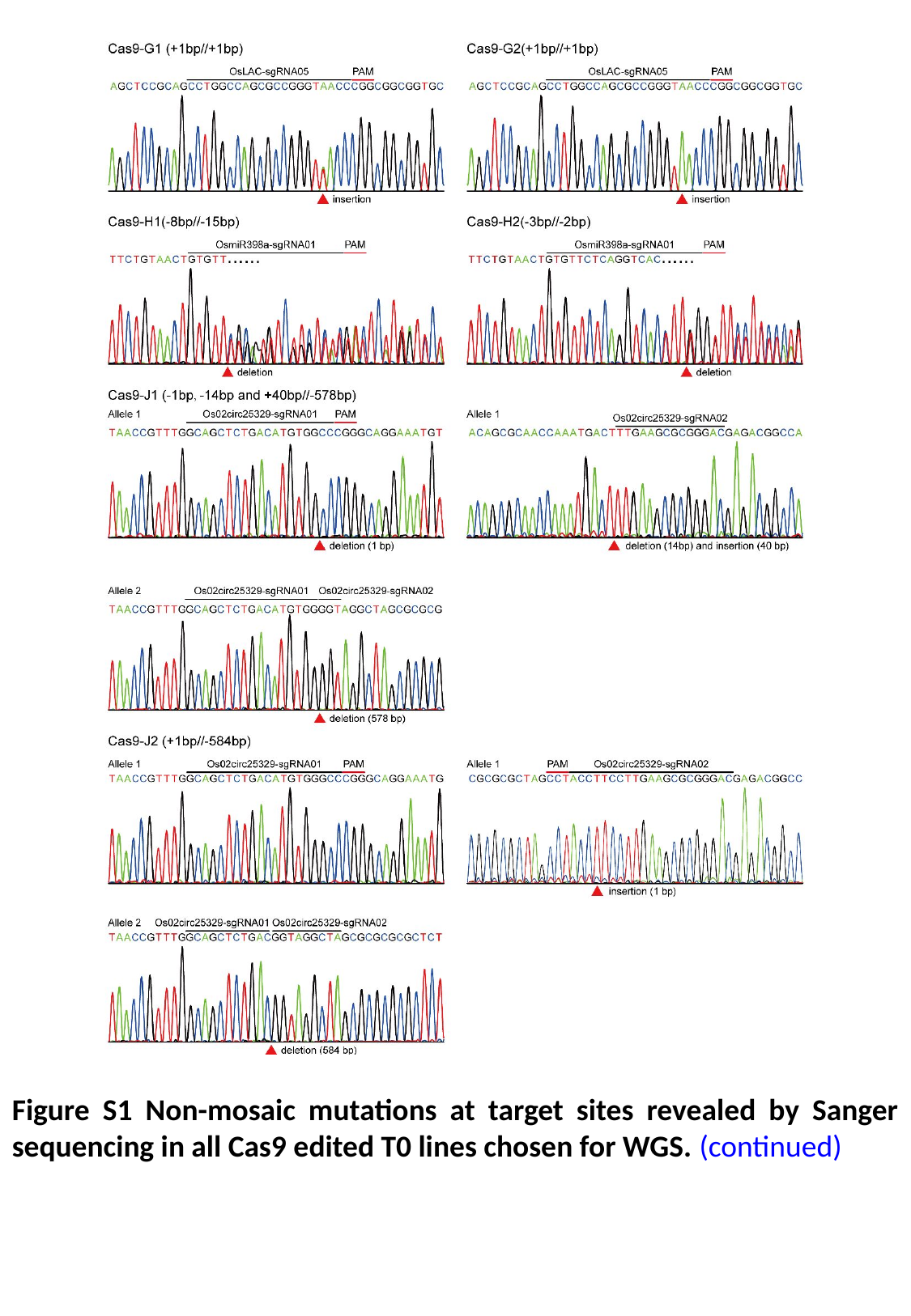

Figure S1 Non-mosaic mutations at target sites revealed by Sanger sequencing in all Cas9 edited T0 lines chosen for WGS. (continued)

## Slide 3
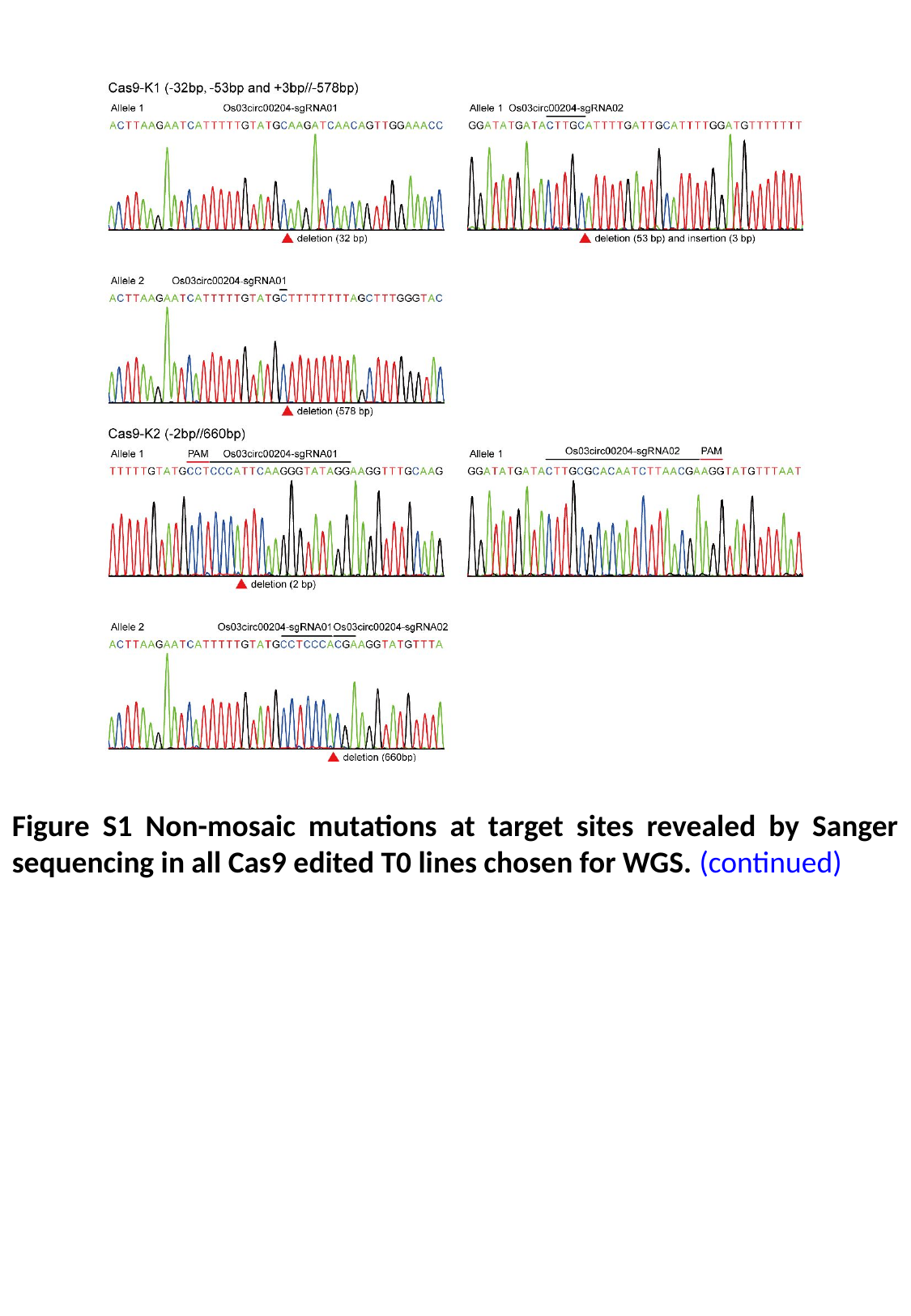

Figure S1 Non-mosaic mutations at target sites revealed by Sanger sequencing in all Cas9 edited T0 lines chosen for WGS. (continued)

## Slide 4
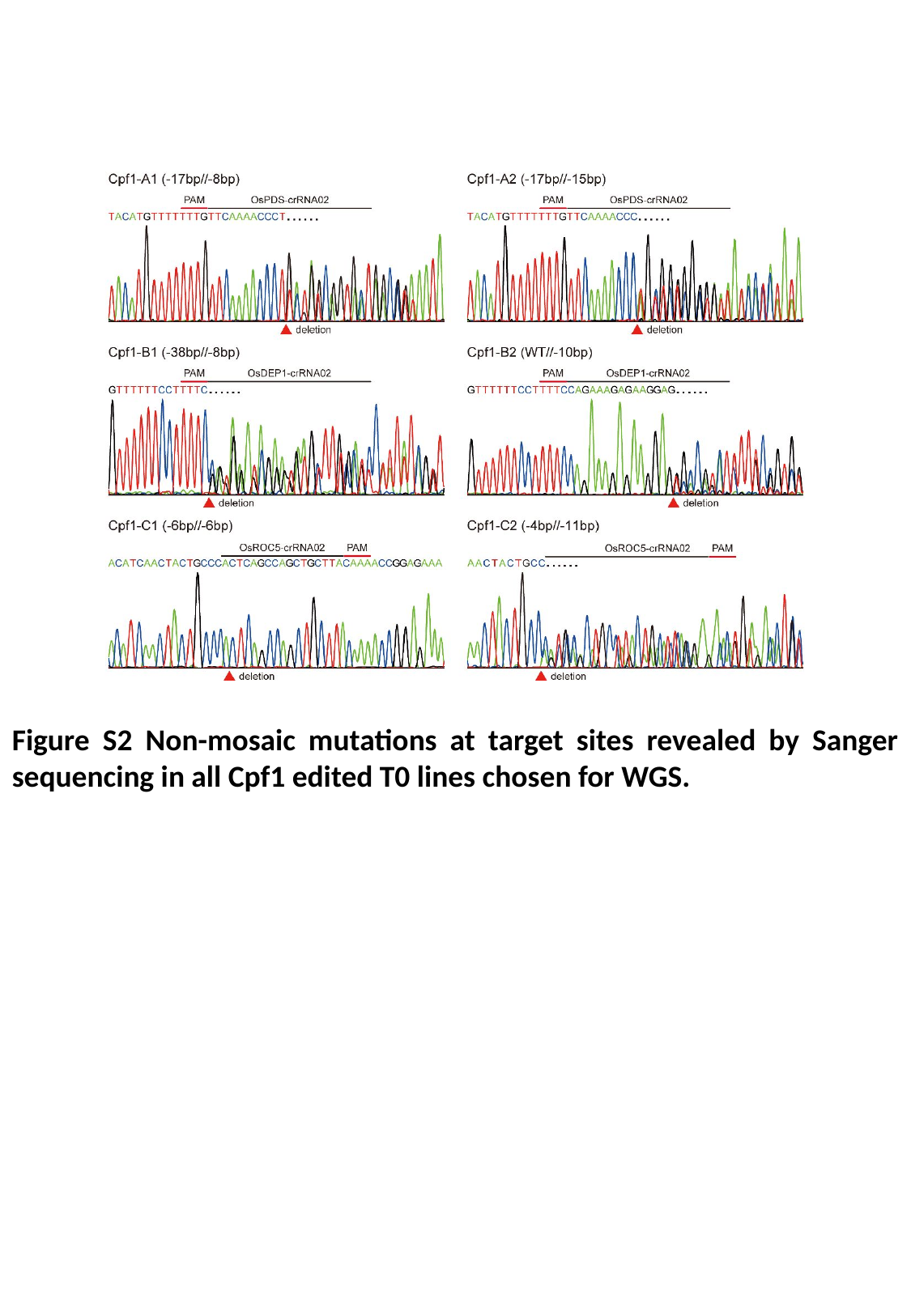

Figure S2 Non-mosaic mutations at target sites revealed by Sanger sequencing in all Cpf1 edited T0 lines chosen for WGS.

## Slide 5
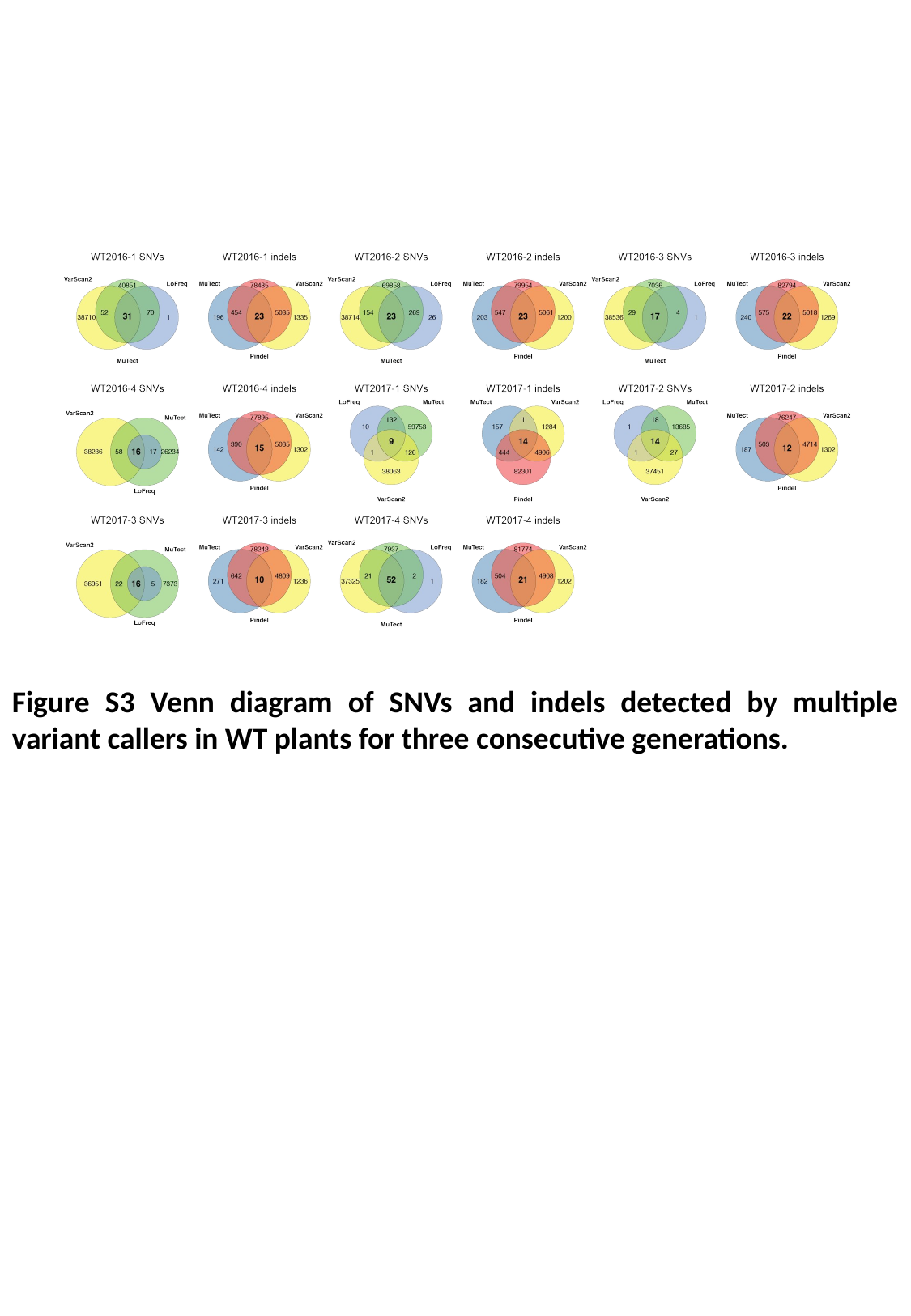

Figure S3 Venn diagram of SNVs and indels detected by multiple variant callers in WT plants for three consecutive generations.

## Slide 6
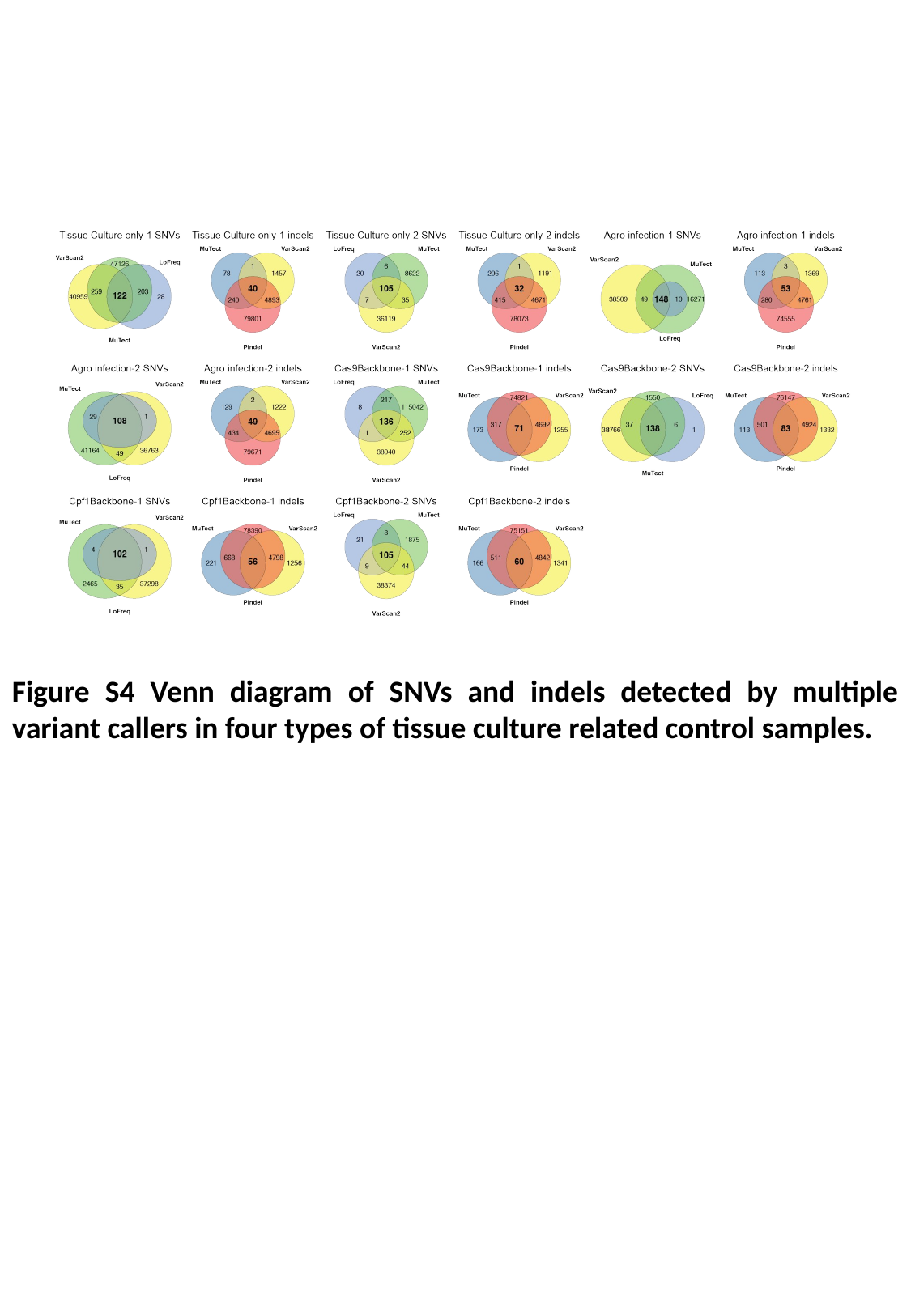

Figure S4 Venn diagram of SNVs and indels detected by multiple variant callers in four types of tissue culture related control samples.

## Slide 7
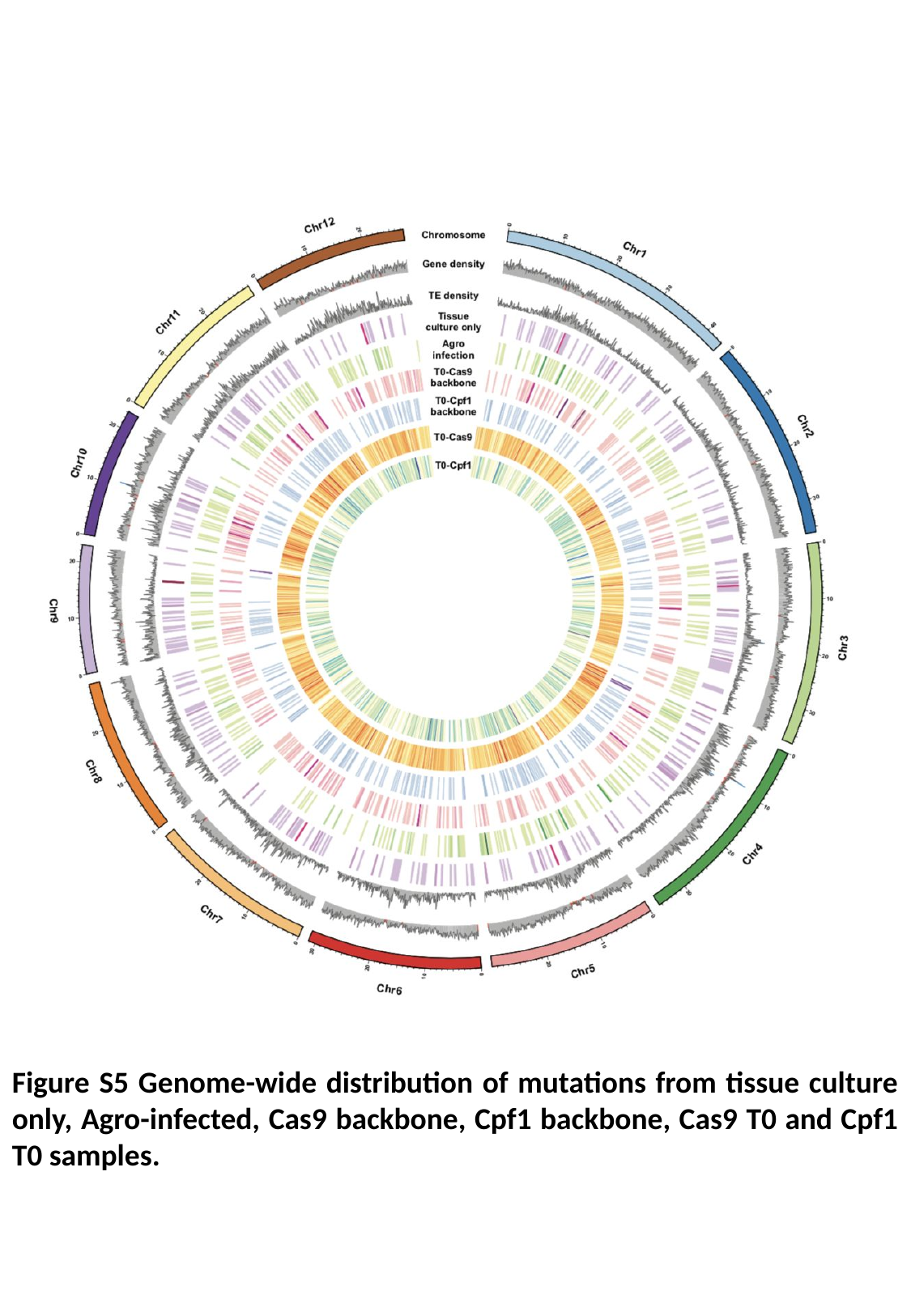

Figure S5 Genome-wide distribution of mutations from tissue culture only, Agro-infected, Cas9 backbone, Cpf1 backbone, Cas9 T0 and Cpf1 T0 samples.

## Slide 8
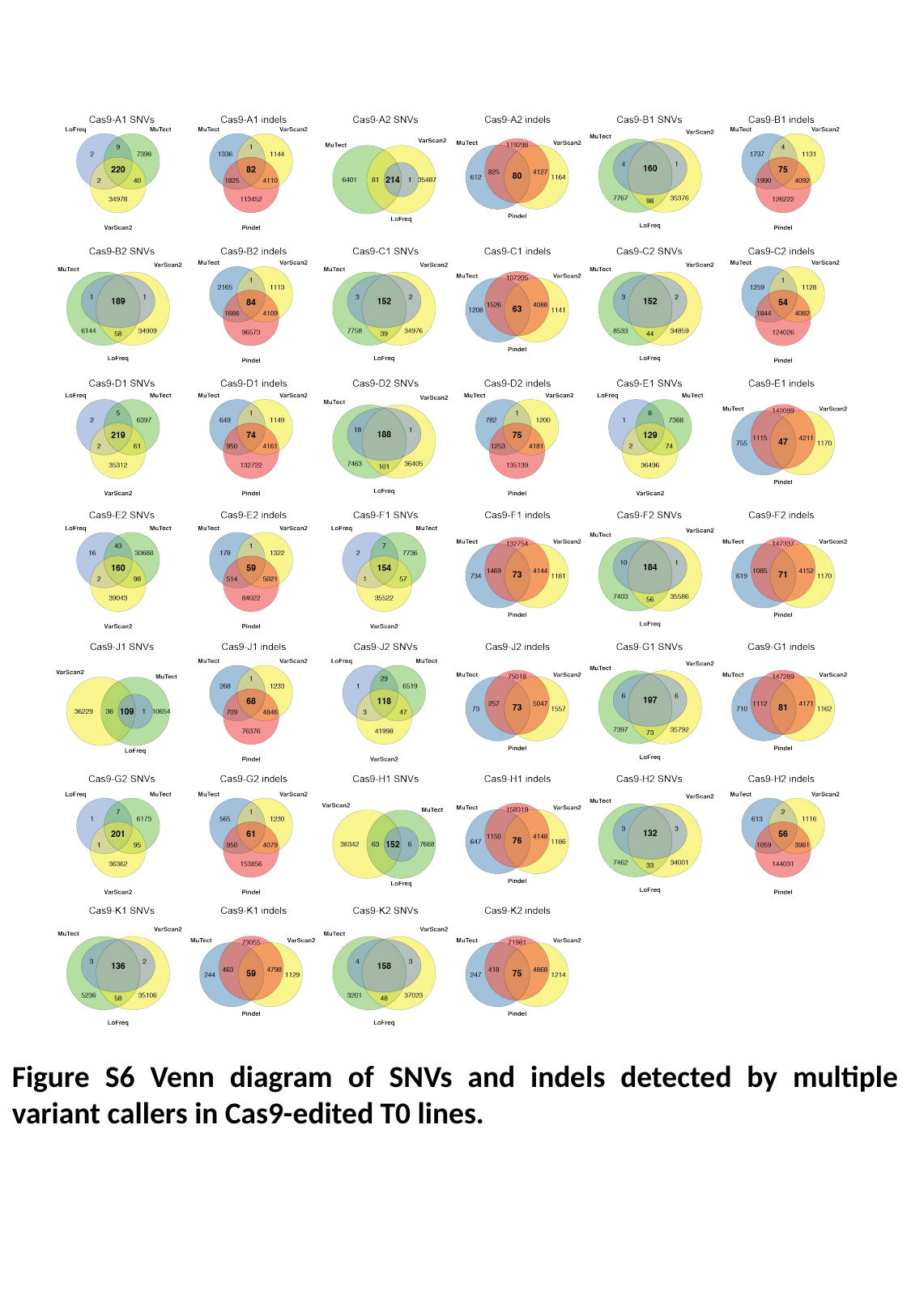

Figure S6 Venn diagram of SNVs and indels detected by multiple variant callers in Cas9-edited T0 lines.

## Slide 9
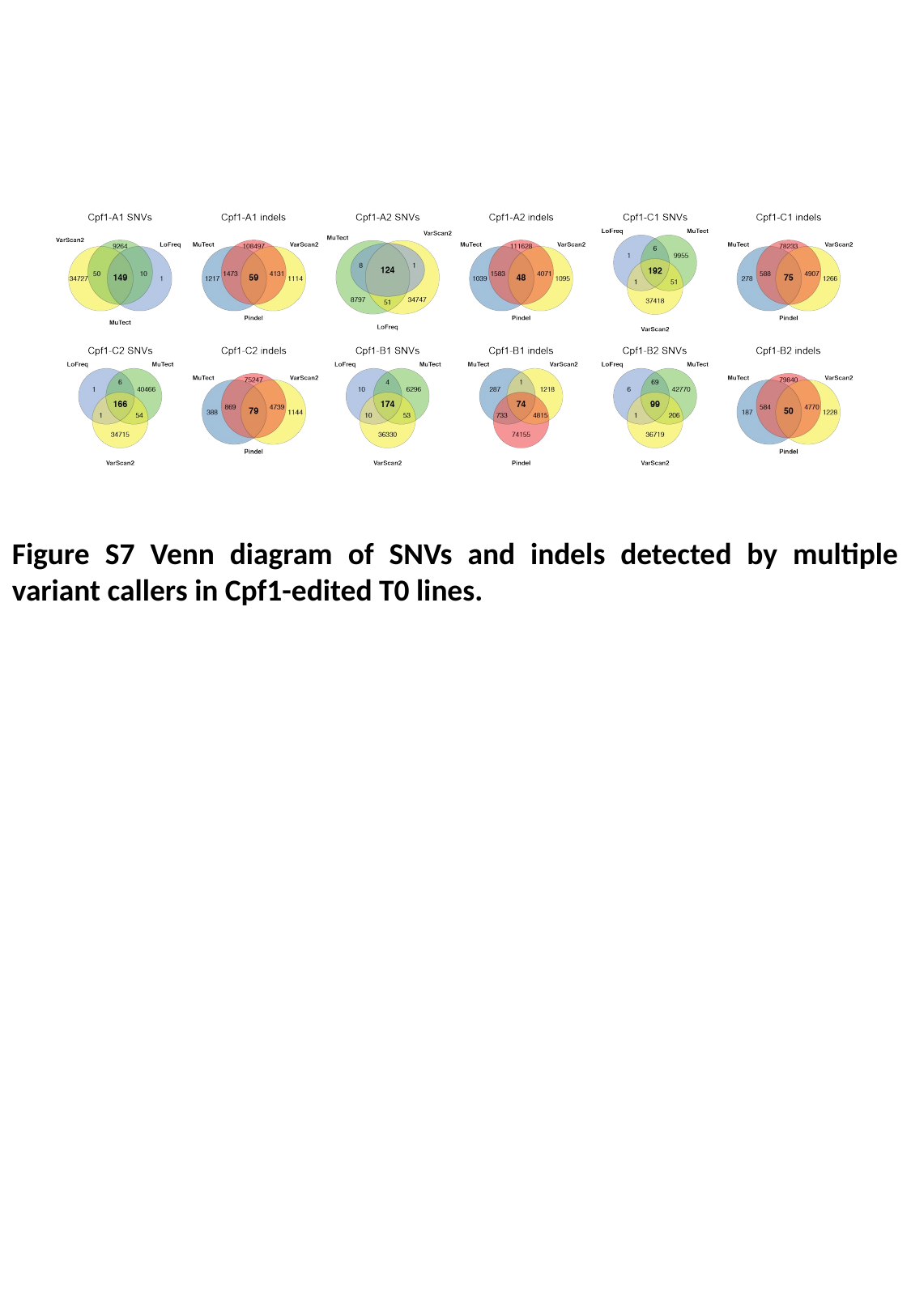

Figure S7 Venn diagram of SNVs and indels detected by multiple variant callers in Cpf1-edited T0 lines.

## Slide 10
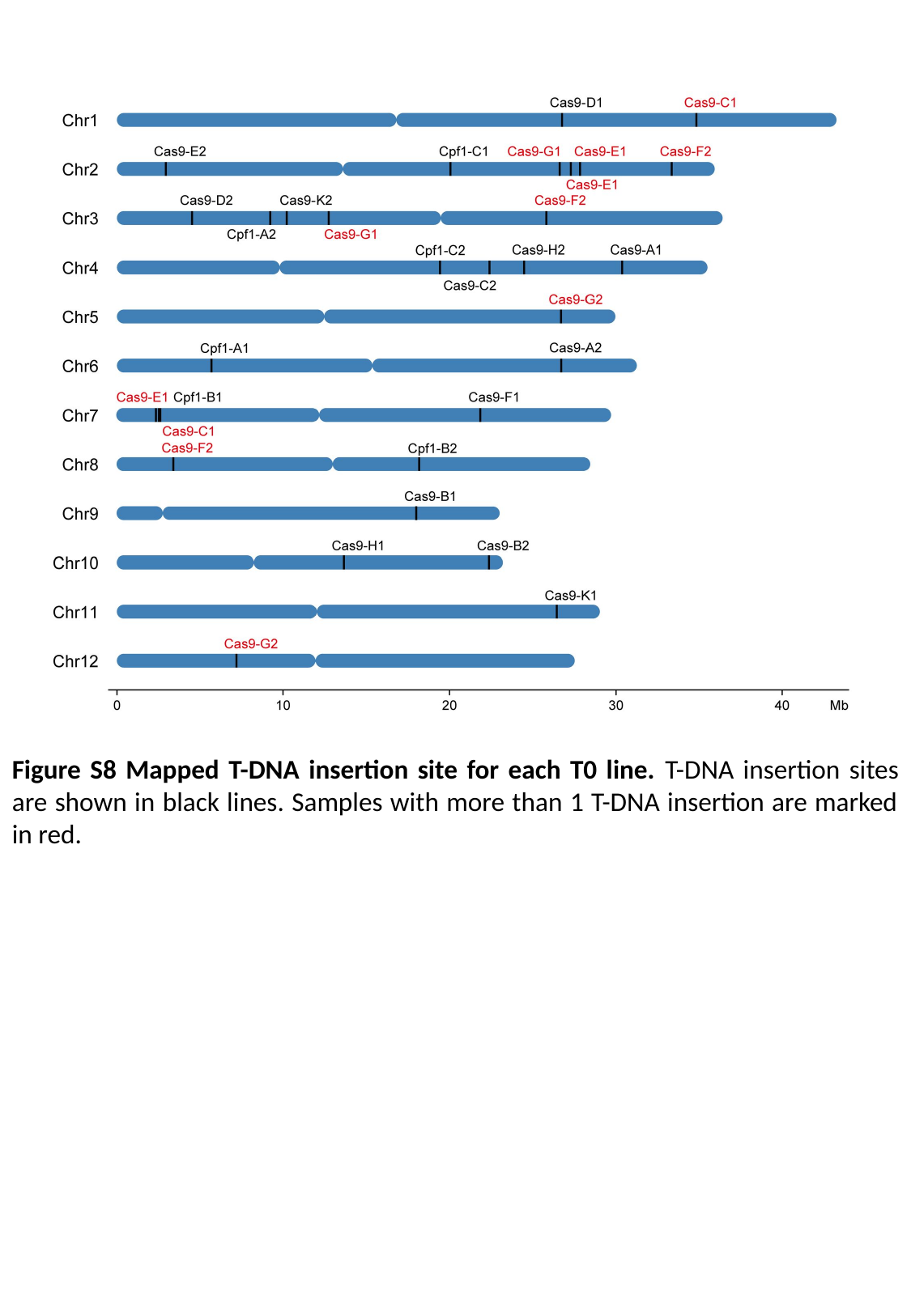

Figure S8 Mapped T-DNA insertion site for each T0 line. T-DNA insertion sites are shown in black lines. Samples with more than 1 T-DNA insertion are marked in red.

## Slide 11
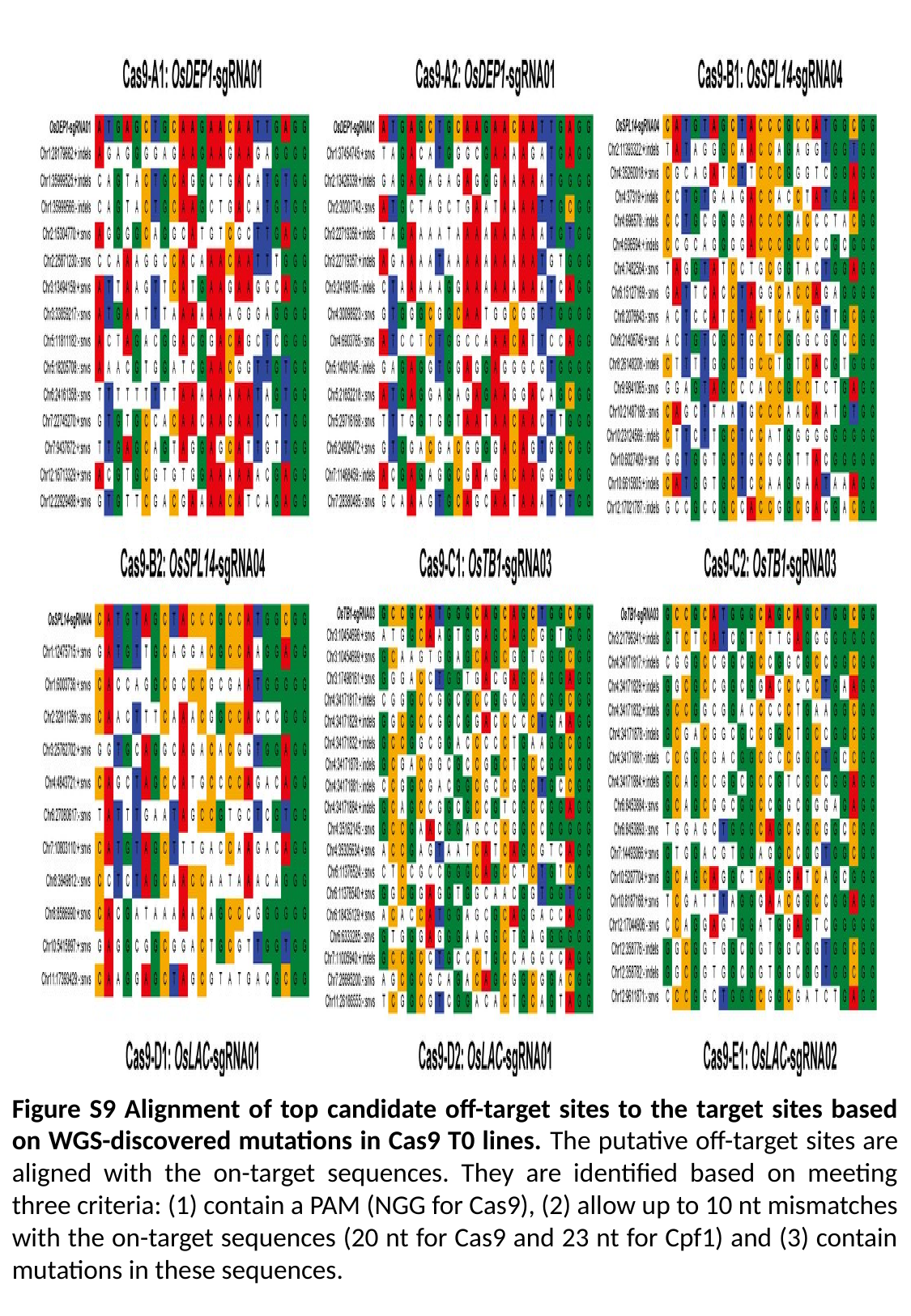

Figure S9 Alignment of top candidate off-target sites to the target sites based on WGS-discovered mutations in Cas9 T0 lines. The putative off-target sites are aligned with the on-target sequences. They are identified based on meeting three criteria: (1) contain a PAM (NGG for Cas9), (2) allow up to 10 nt mismatches with the on-target sequences (20 nt for Cas9 and 23 nt for Cpf1) and (3) contain mutations in these sequences.
