# Supplementary material for: A large-scale whole-genome sequencing analysis reveals highly specific genome editing by both Cas9 and Cpf1 (Cas12a) nucleases in rice
Source: Genome Biol. 2018 Jul 4;19:84. doi: 10.1186/s13059-018-1458-5 (PMC6031188; doi:10.1186/s13059-018-1458-5)
Supplement: Supplementary file 3 — Figures S10–S18. Supplemental Figures-part II. (PPTX 13267 kb) [file 13059_2018_1458_MOESM3_ESM.pptx]

## Slide 1
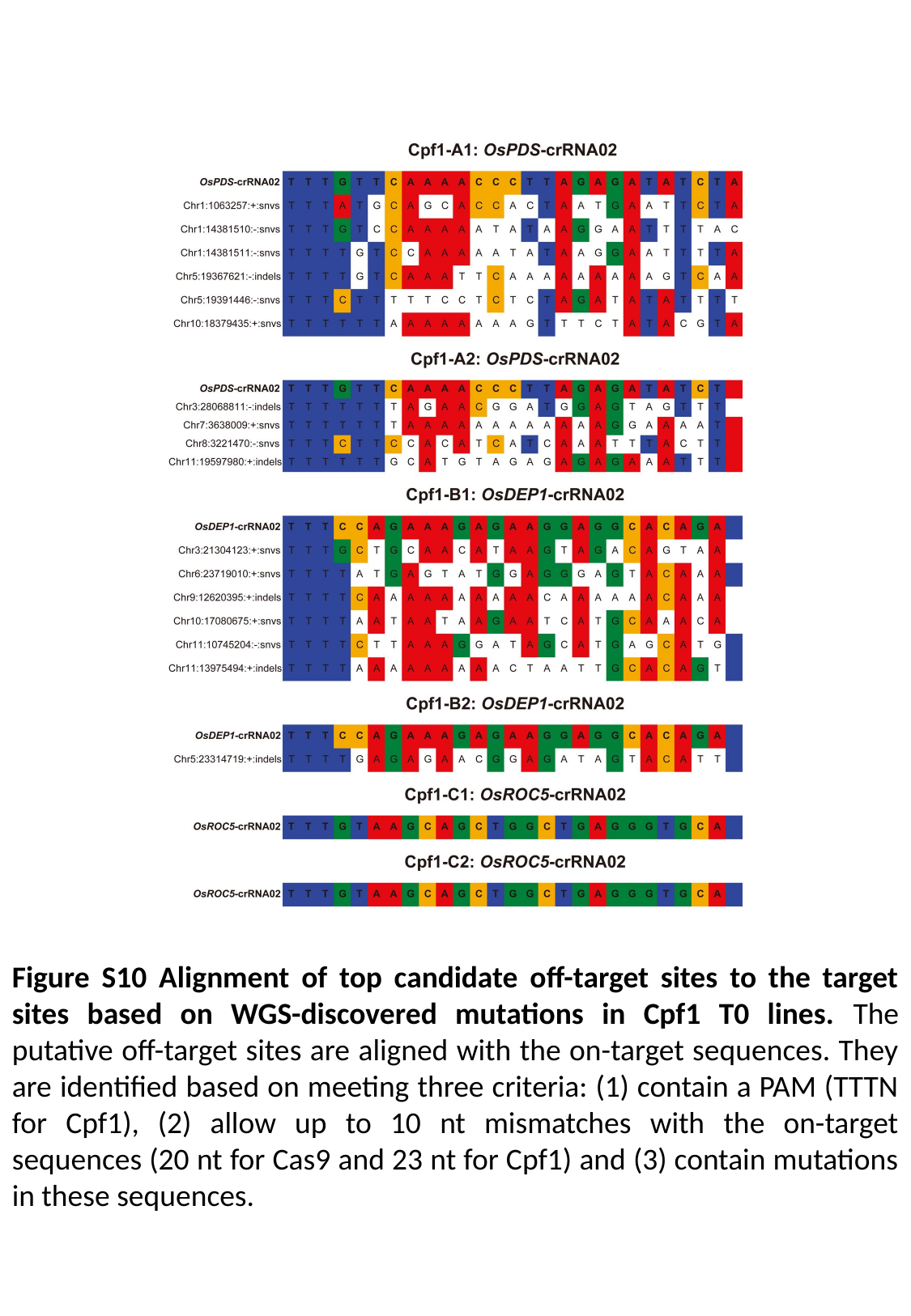

Figure S10 Alignment of top candidate off-target sites to the target sites based on WGS-discovered mutations in Cpf1 T0 lines. The putative off-target sites are aligned with the on-target sequences. They are identified based on meeting three criteria: (1) contain a PAM (TTTN for Cpf1), (2) allow up to 10 nt mismatches with the on-target sequences (20 nt for Cas9 and 23 nt for Cpf1) and (3) contain mutations in these sequences.

## Slide 2
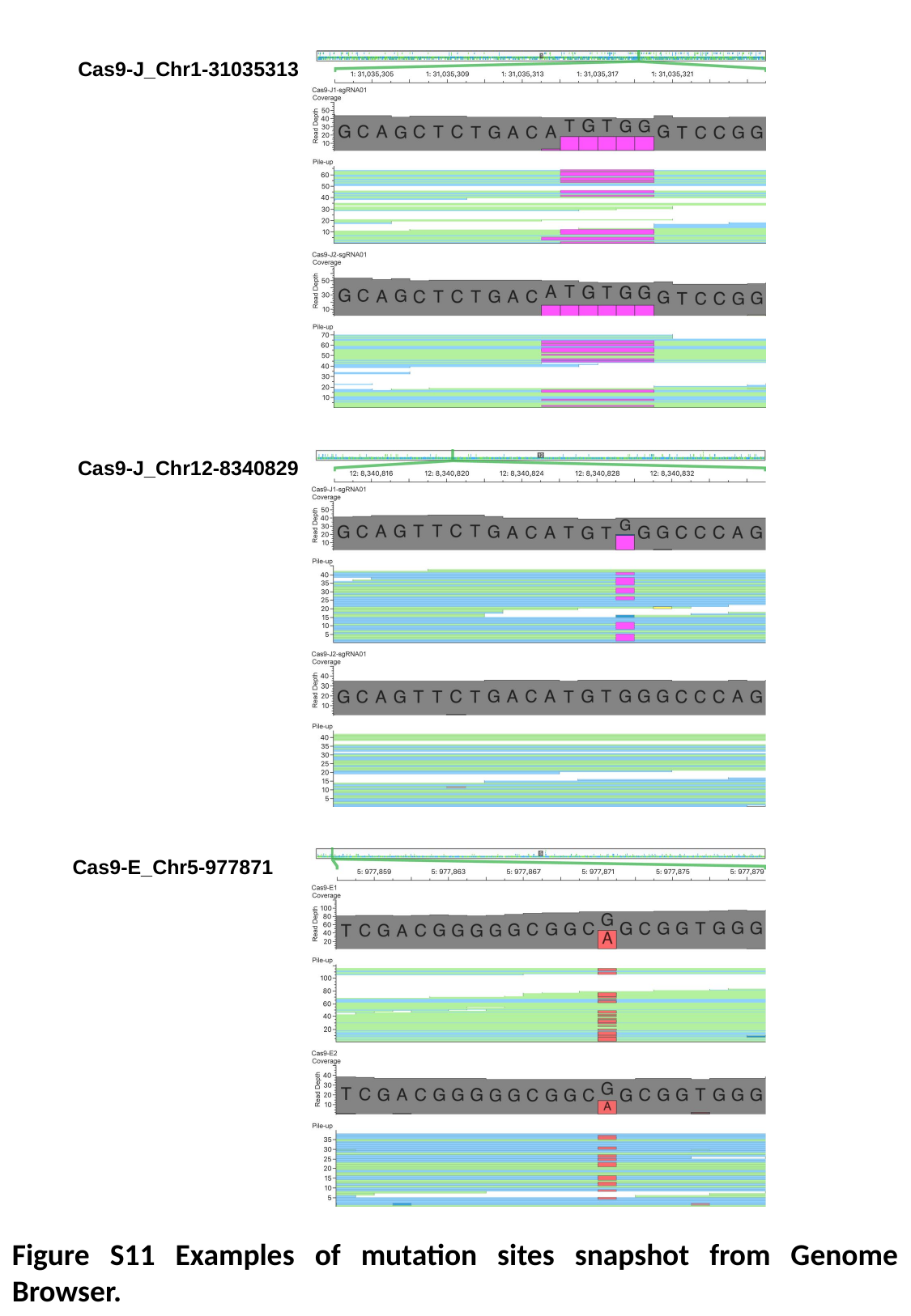

Cas9-J_Chr1-31035313
Cas9-J_Chr12-8340829
Cas9-E_Chr5-977871
Figure S11 Examples of mutation sites snapshot from Genome Browser.

## Slide 3
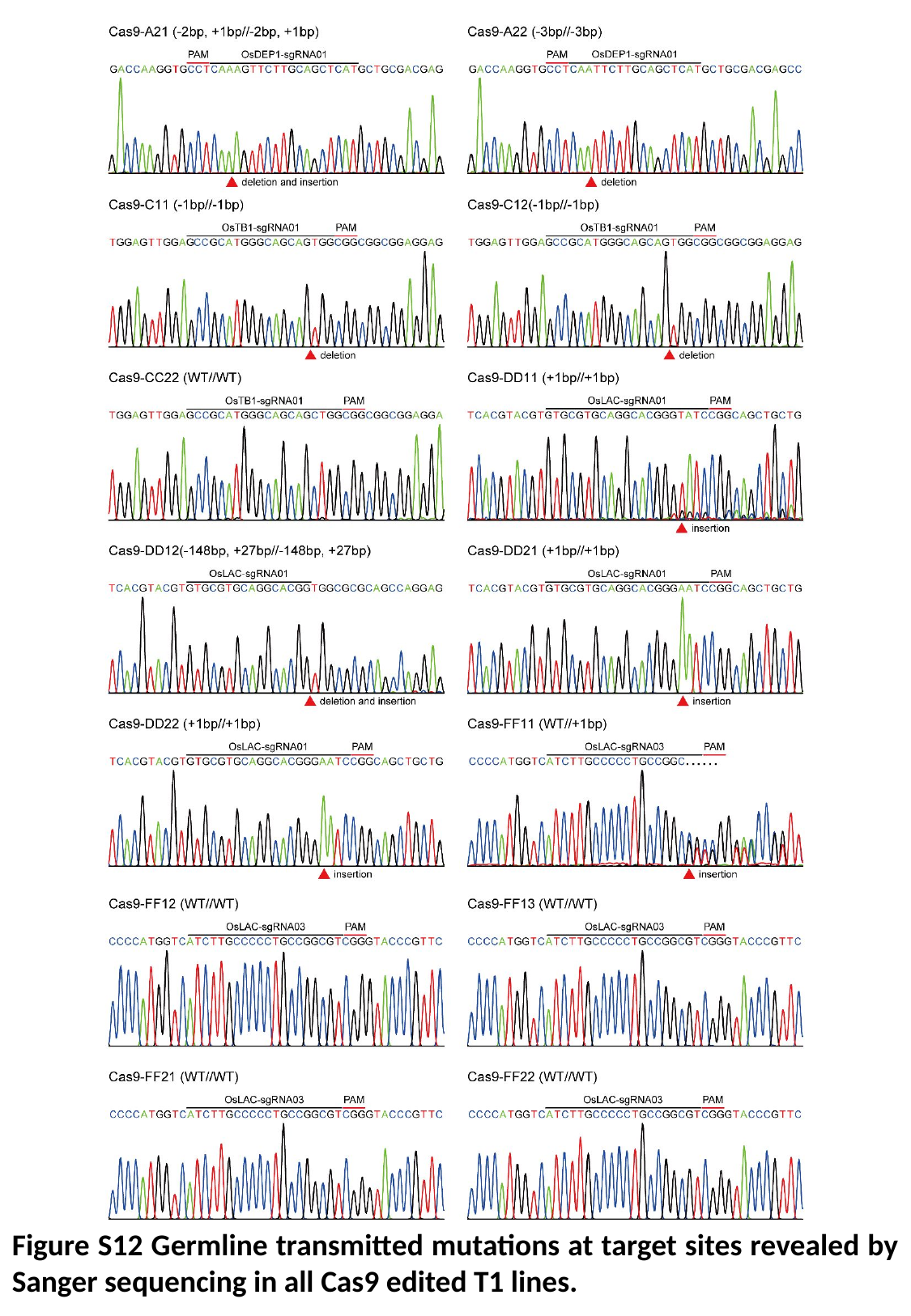

Figure S12 Germline transmitted mutations at target sites revealed by Sanger sequencing in all Cas9 edited T1 lines.

## Slide 4
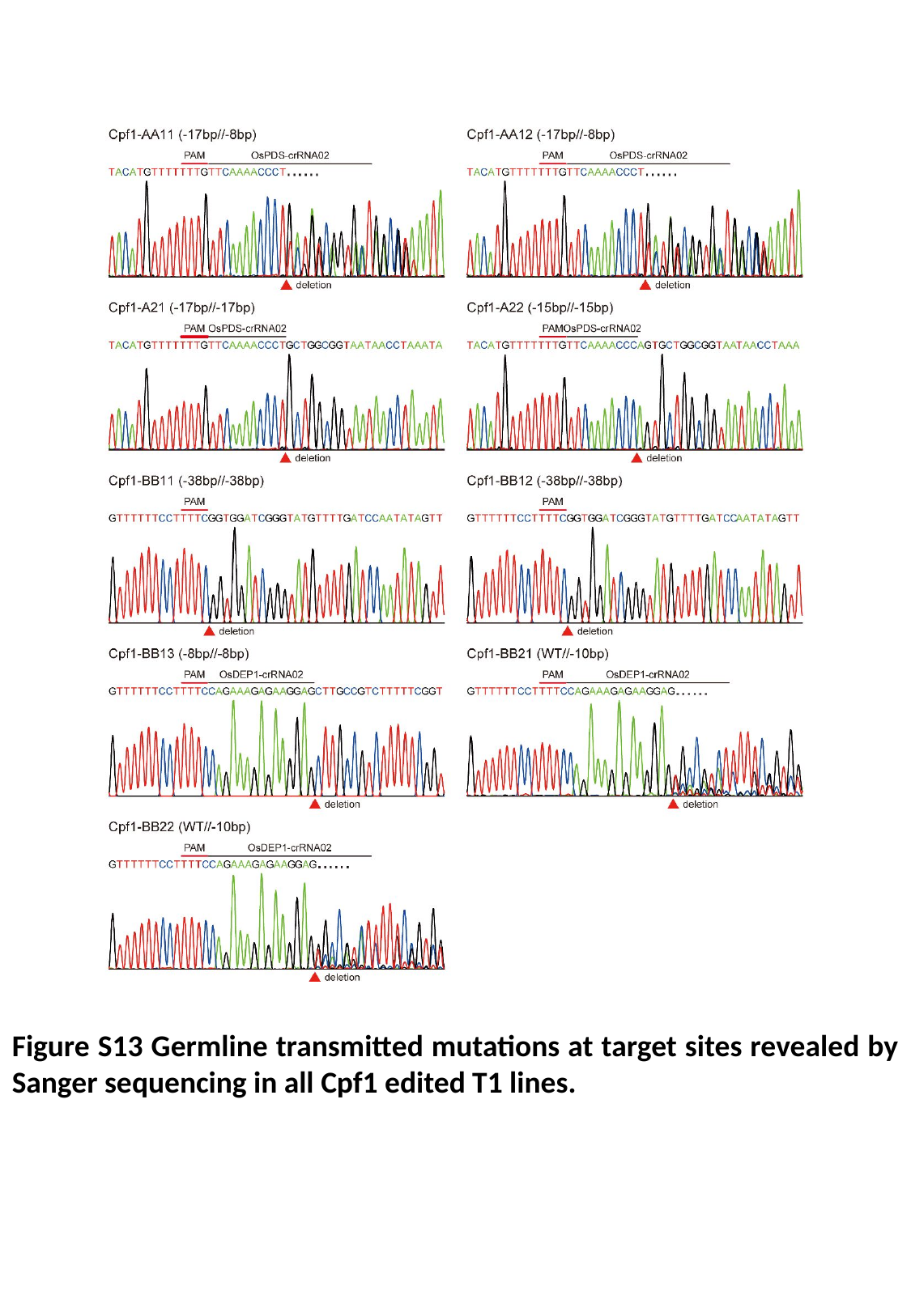

Figure S13 Germline transmitted mutations at target sites revealed by Sanger sequencing in all Cpf1 edited T1 lines.

## Slide 5
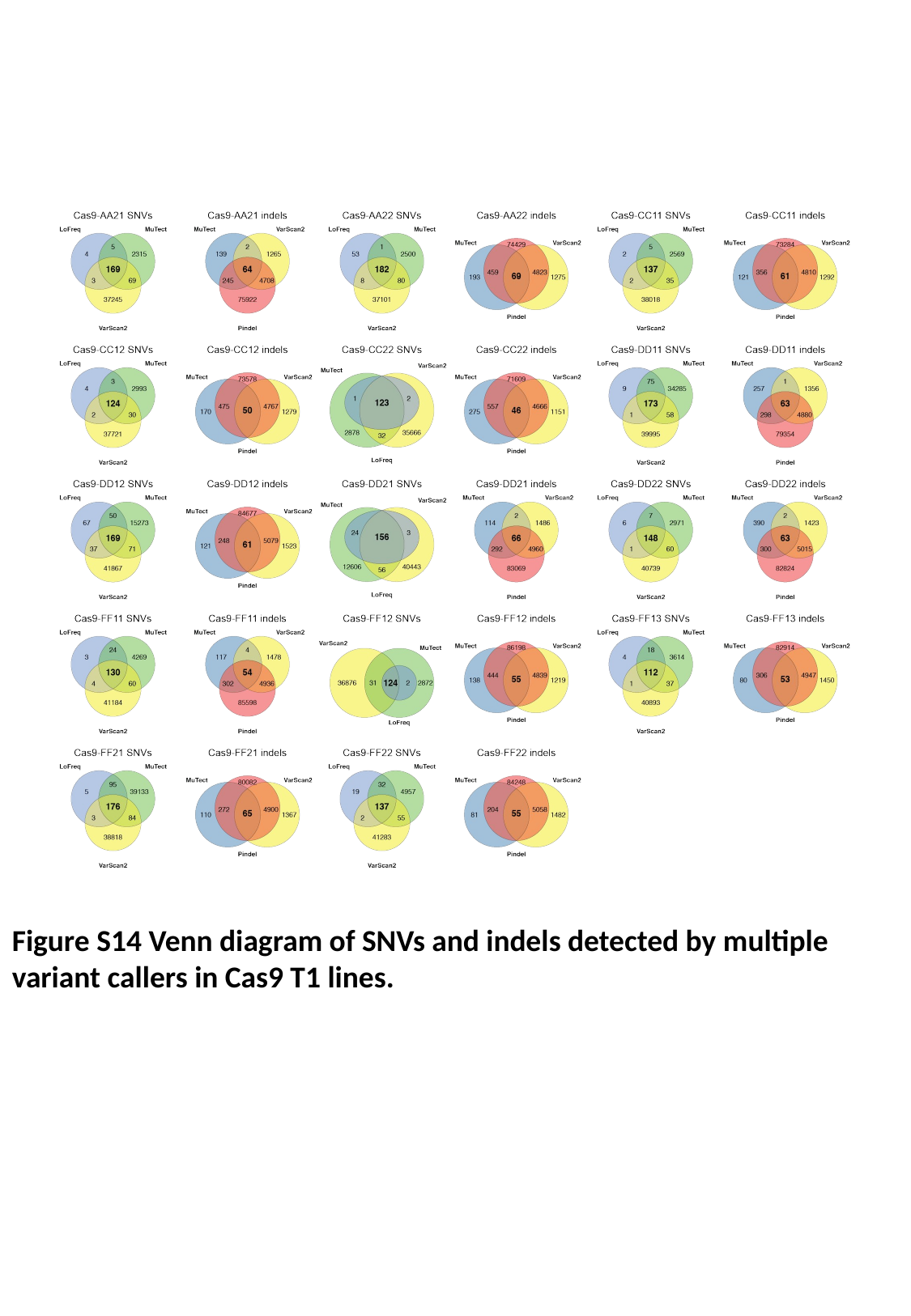

Figure S14 Venn diagram of SNVs and indels detected by multiple variant callers in Cas9 T1 lines.

## Slide 6
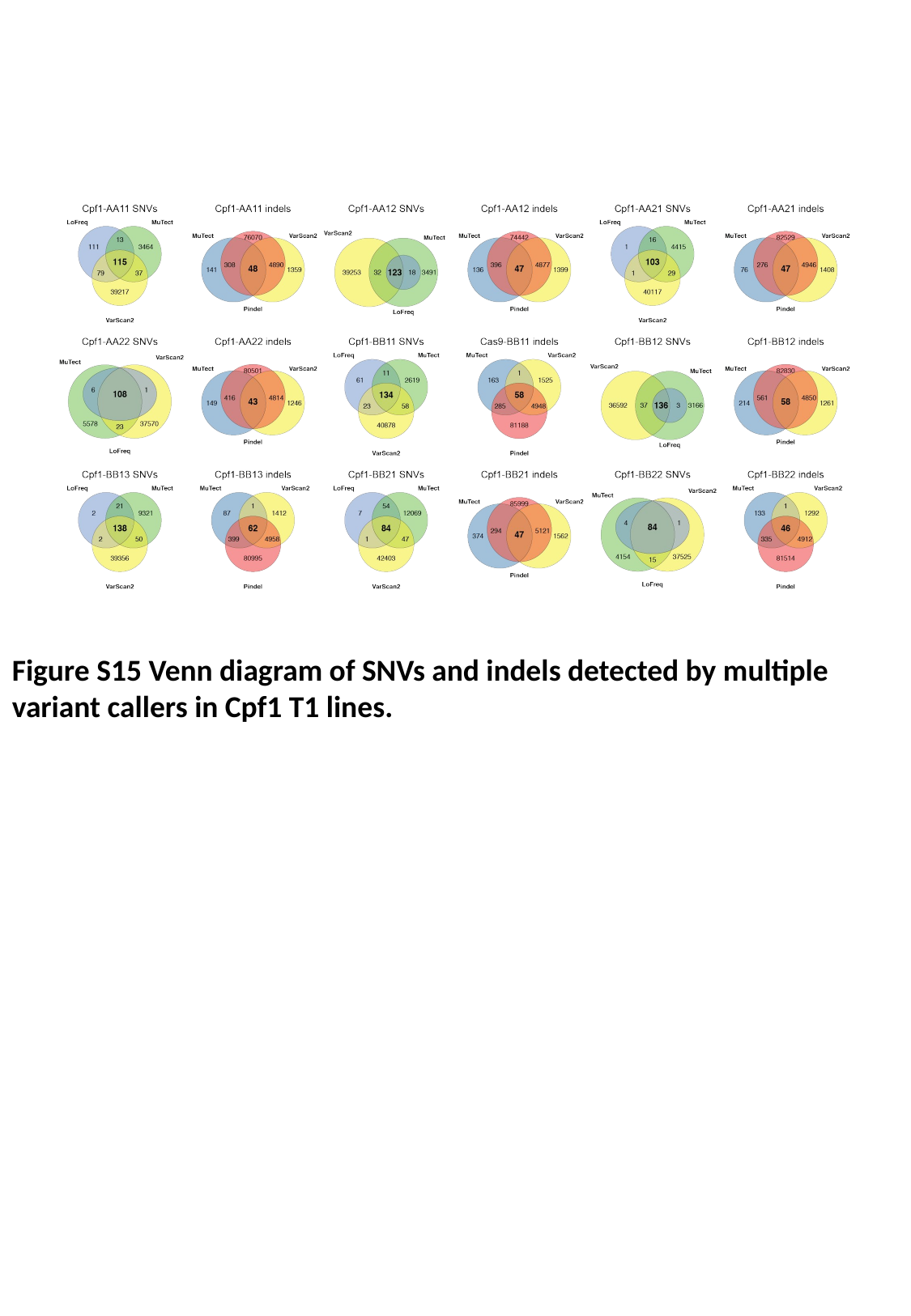

Figure S15 Venn diagram of SNVs and indels detected by multiple variant callers in Cpf1 T1 lines.

## Slide 7
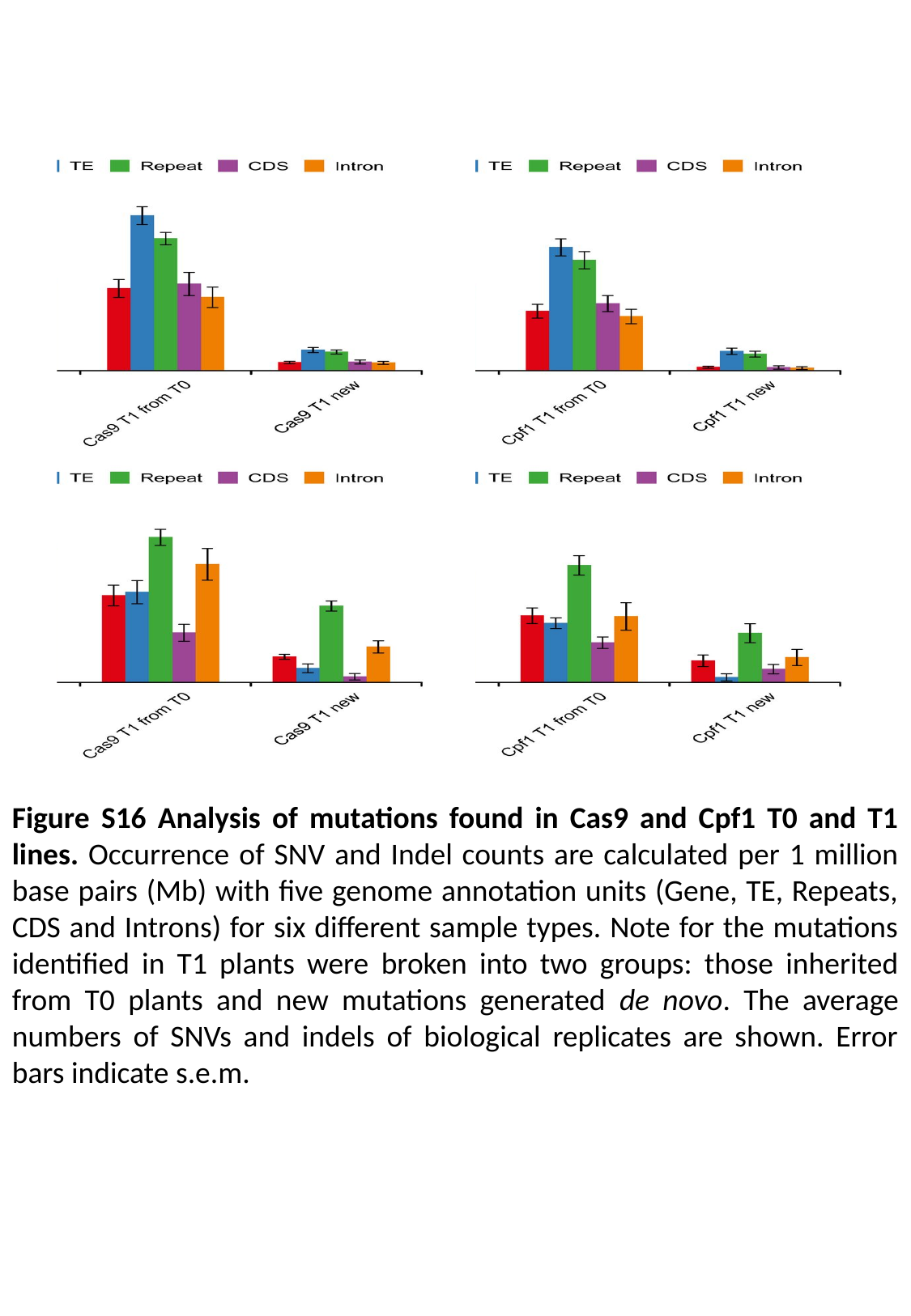

Figure S16 Analysis of mutations found in Cas9 and Cpf1 T0 and T1 lines. Occurrence of SNV and Indel counts are calculated per 1 million base pairs (Mb) with five genome annotation units (Gene, TE, Repeats, CDS and Introns) for six different sample types. Note for the mutations identified in T1 plants were broken into two groups: those inherited from T0 plants and new mutations generated de novo. The average numbers of SNVs and indels of biological replicates are shown. Error bars indicate s.e.m.

## Slide 8
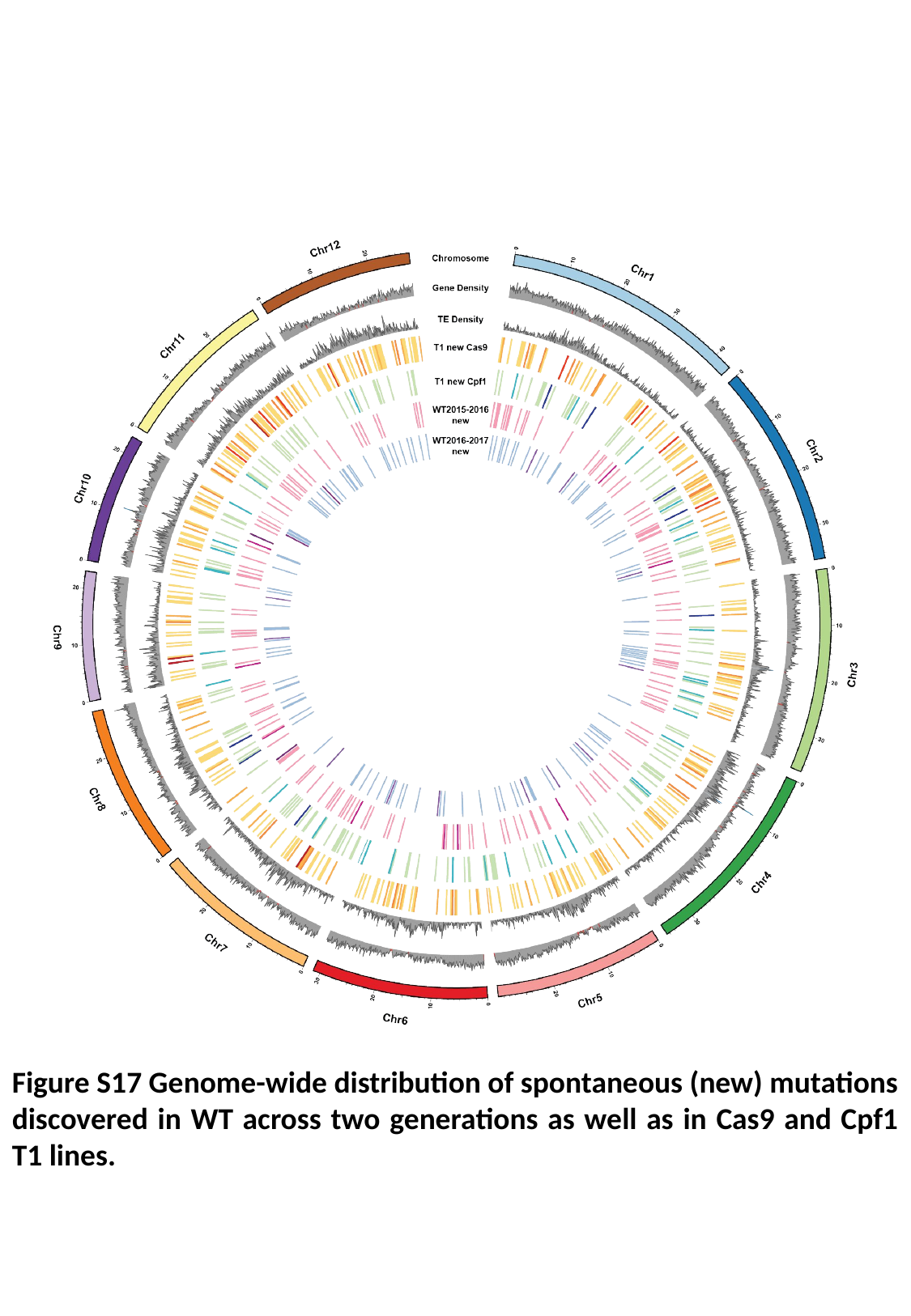

Figure S17 Genome-wide distribution of spontaneous (new) mutations discovered in WT across two generations as well as in Cas9 and Cpf1 T1 lines.

## Slide 9
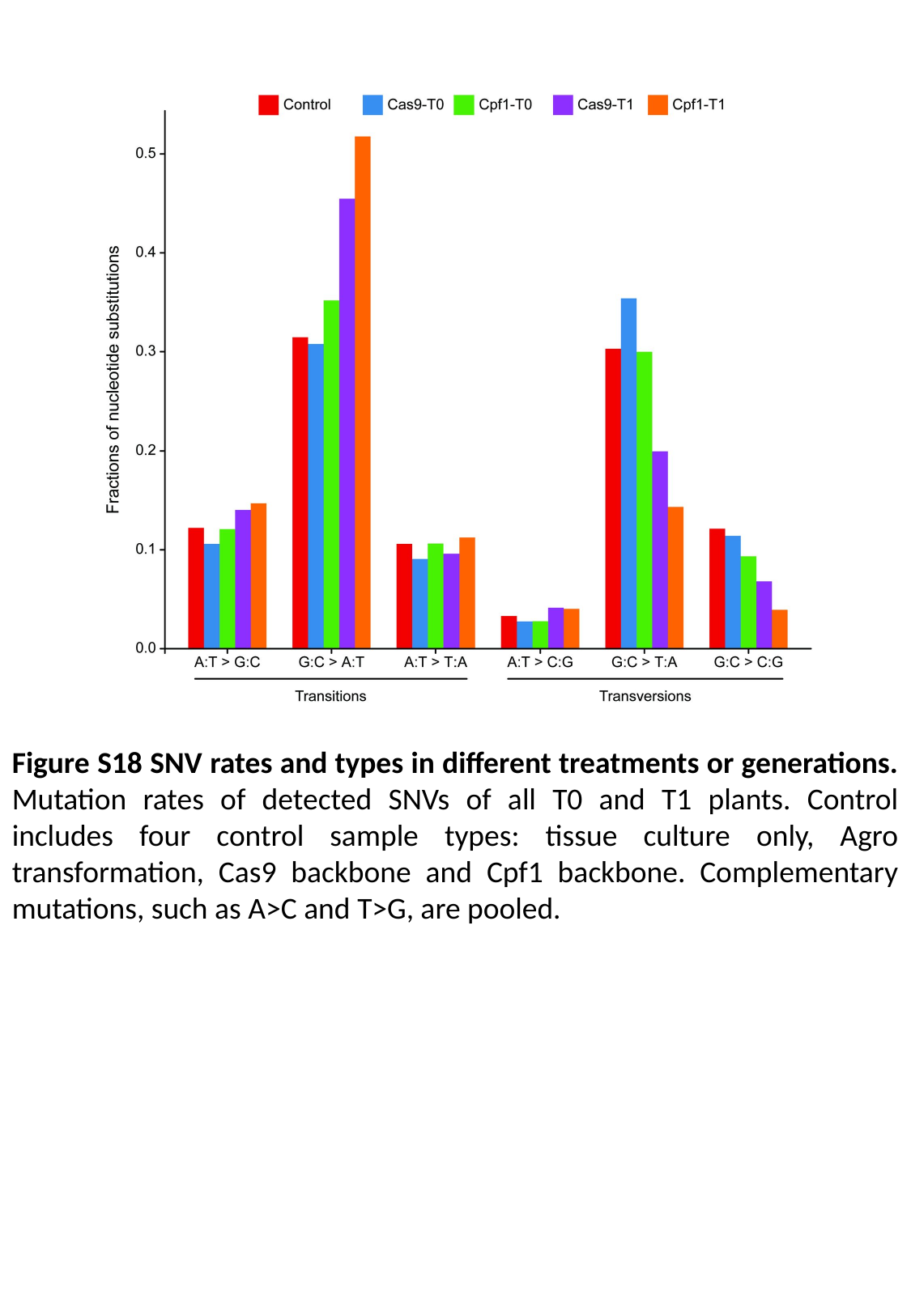

Figure S18 SNV rates and types in different treatments or generations. Mutation rates of detected SNVs of all T0 and T1 plants. Control includes four control sample types: tissue culture only, Agro transformation, Cas9 backbone and Cpf1 backbone. Complementary mutations, such as A>C and T>G, are pooled.
